# Effectiveness of brace treatment for adolescent idiopathic scoliosis

**DOI:** 10.1186/1748-7161-10-S2-S12

**Published:** 2015-02-11

**Authors:** Toru Maruyama, Yosuke Kobayashi, Makoto Miura, Yusuke Nakao

**Affiliations:** 1 Department of Orthopaedic Surgery, Saitama Medical Center, Saitama Medical University, 1981 Kamoda, Kawagoe, Saitama, 350-8550 Japan

## Abstract

**Objectives:**

Effectiveness of brace treatment for adolescent idiopathic scoliosis (AIS) was demonstrated by the BrAIST study in 2013. Objectives of this study were to confirm its effectiveness by analyzing our results and to clarify the factors affecting the results of the treatment.

**Materials and methods:**

According to the Scoliosis Research Society AIS brace studies standardization criteria, patients with age 10 years or older, Risser 0 to II, less than 1 year post-menarche, curve magnitude 25 to 40 degrees before brace treatment and who received no prior treatment were included in the study. At skeletal maturity, the rate of the patients whose curve was stabilized, exceeded 45 degrees, and who were recommended or underwent surgery were investigated. Additionally, initial correction rate by the brace and factors affecting the results were investigated.

**Results:**

A total of 33 patients (27 females and 6 males) could be followed-up until their skeletal maturity and included in the analysis. An average age was 11.9 years, average Cobb angle was 30.8°, and Risser sign was 0 in 13 patients, I in 5, and II in 15 patients before treatment. There were 13 thoracic curves, 14 thoracolumbar or lumbar curves, and 6 double curves. Initial correction rate by the brace was 53.8% for the total curves. In terms of curve pattern, 34.4% for thoracic curve, 73.9% for thoracolumbar or lumbar curve, and 48.8% for double curve.

After an average follow-up period of 33 months, 8 patients improved in more than 6 degrees, change of 17 patients were within 6 degrees, and 8 progressed in more than 6 degrees. Therefore, totally, 76% (25/33) of the curves were stabilized by the treatment. Four curves (12%) exceeded 45 degrees and one patient (3%) underwent surgery. Our results were better than the reported natural history. Factors that affected the results were hump degree before treatment and initial correction rate by the brace.

**Conclusions:**

76% of the curve with AIS could be stabilized by brace treatment. Brace treatment was effective for treatment of AIS. Factors affecting the results were hump degrees and initial correction rate.

## Background

Effectiveness of brace treatment for AIS was demonstrated by BrAIST study in 2013 [1]. Purpose of this study was to certify the effectiveness of brace treatment by analyzing our treatment results and to clarify the factors affecting the results of the treatment.

## Materials and methods

With Institutional Review Board of Saitama Medical Center approval, we retrospectively reviewed the prospective database that started in April 2007. According to the SRS brace studies standardization criteria [2], AIS patients with age 10 years or older, Risser 0 to 2, pre-menarche or less than 1 year post-menarche, curve magnitude 25 to 40 degrees before brace treatment and who received no prior treatment were included in the study. The rate of the patients whose curve was stabilized, exceeded 45 degrees, and who were recommended or underwent surgery at skeletal maturity was investigated. Stabilization of the curve was defined as the curve not progressed in more than 6 degrees at skeletal maturity comparing with the pre-treatment Cobb angle.

Additionally, initial correction rate by the brace was calculated comparing pre-treatment Cobb angle and first in-brace Cobb angle. Factors affecting the results were analyzed by single variable analysis. In the statistical analysis, Student t-test was used for continuous variables and chi-square test was used for discontinuous variables. A p-value of less than 0.05 was considered to be significant.

## Description of the brace system and treatment protocol

**Figure 1:**
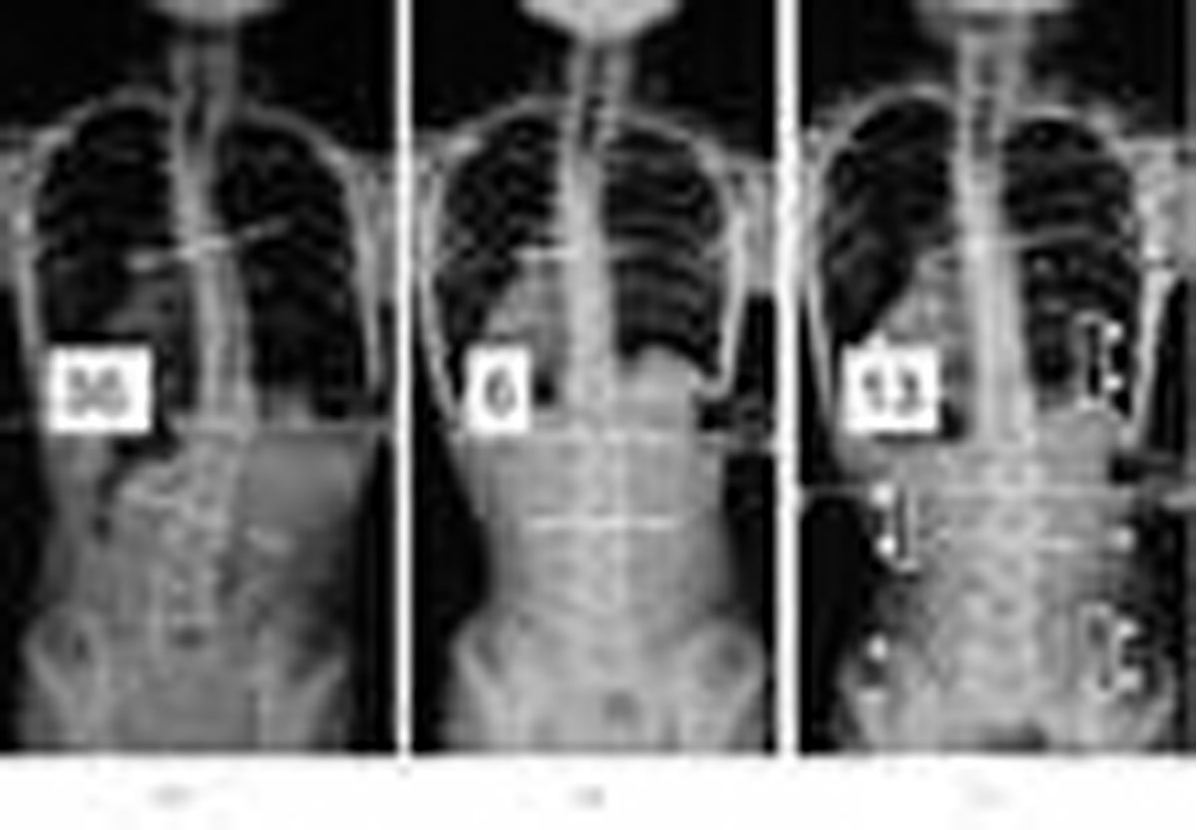
**a: before treatment, b: first in-brace radiograph at the time of trial fitting, c: second in-brace radiograph after 2 months**. Informed consent was obtained from the patient for the image(s) used in this study.

We have been using Rigo-Chêneau type brace (Figure 1). When brace treatment is indicated, plaster molding is done with the patient in the standing position. The patient is instructed to shift her trunk to the concavity of the curve while molding in order to locate her/his thoracic cage properly on the pelvis. One week after, trial fitting is conducted and the first in-brace radiograph is taken. The brace is adjusted and completed one more week after. Two to three months after completion of the brace, second in-brace radiograph is taken and the brace is adjusted if necessary.

## Results

A total of 33 patients were included in the analysis. Patients’ demographic characteristics were shown in Table 1.

**Table 1:** Patients’ demographic characteristics

Gender (number)	Female	27
	Male	6

Age (year)	11.9 ± 1.2	

Cobb angle (degree)	30.8 ± 4.7	

Risser sign (number)	0	13

	I	5

	II	15

Curve pattern (number)	Thoracic	13

	Thoracolumbar or lumbar	14

	Double major	6

Initial correction rate of the total curves were 53.8%. In terms of curve pattern, 34.4% for thoracic curve, 73.9% for thoracolumbar or lumbar curve, and 44.8% for double curve.

After an average follow-up period of 33 months, all the patients had reached skeletal maturity. No patients were dropped out or lost to follow-up during the study period. Of 33 patients, 8 improved in more than 6 degrees, change of 17 patients were within 6 degrees, and 8 progressed in more than 6 degrees. Therefore, totally 75.8% (25/33) of the curves were stabilized by the treatment. Four curves (12%) exceeded 45 degrees and one patient (3%) underwent surgery.

Factors affecting the results, whether the curve was improved or progressed, were hump degrees before treatment and initial correction rate by the brace (Table 2). In other words, better results were predicted for patients with smaller hump degrees, or the patients with better initial correction rate. Other factors had no statistically significant effect on the results.

**Table 2:** Factors affecting the results

	Improved (n=8)	Progressed (n=8)	
Gender	Female 6, Male 2	Female 6, Male 2	>0.9999

Age	11.5 ± 1.4	12.1 ± 1.1	0.3447

Risser sign	0 2, I 1, II 5	0 6, I 1, II 1	0.0819

Curve pattern	T 2, TL or L 5, D 1	T 4, TL or L 1, D 3	0.0984

Cobb (degree)	29.4 ± 4.5	31.9 ± 4.6	0.2911

Hump (degree)	5.8 ± 3.3	9.8 ± 2.8	0.0204 *^1^

Correction (%)	83.4 ± 30.2	37.6 ± 24.1	0.0048 *^1^

*^1^: statistically significant

## Discussion

Results of this study showed that 76% of the curves with AIS patients who met the SRS inclusion criteria were stabilized by the brace treatment. These results were compared with natural history and other brace studies using the SRS standardization criteria (Table 3).

**Table 3:** Comparison of our results, natural history, and other brace studies

Author	Year	Treatment	N	Stabilize	45° <	Surgery
Maruyama	2014	Rigo-Chêneau	33	76%	12%	3%

Bunnell [3]	1986	Natural history	NA	33%	NA	NA

Nachemson[4]	1995	Natural history	129	34%	NA	NA

Janicki[5]	2007	TLSO	48	15%	63%	79%

		Providence	35	31%	43%	60%

Gammon [6]	2010	TLSO	35	60%	28%	23%

		SpineCor	32	53%	20%	19%

Coillard [7]	2007	SpineCor	170	59%	24%	23%

Lee [8]	2012	Charleston	95	84%	16%	8%

Negrini [9]	2009	Various	46	96%	0%	0%

In the natural history, 33% or 34% of the curve had not progressed [3,4], which were much lesser than our results. Other brace studies indicated a wide range of stabilization rate, 15% to 96% [5-9]. Our results were better than most of these studies, even though some of these studies excluded dropped-out patients from analysis.

Results of this study indicated that hump degree and initial correction rate affected the results of the brace treatment. When the number of the patients analyzed will increase, Risser sign or curve pattern may become a predictive factor. Although some studies showed that compliance of the patient was a predictive factor [1,10], we could not include it in the analysis because we had no objective measuring device. Further study will be required with increasing number of the patients and additional factors such as compliance and thoracic kyphosis.

## Conclusion

Seventy-six per cent of the curve with AIS could be stabilized by the brace treatment. Brace treatment was effective for the treatment of AIS. Factors that affected the results of the treatment were hump degree before treatment and initial correction rate by the brace.

This is the extended abstract of IRSSD 2014 program book [11].

## List of abbreviations used

AIS: adolescent idiopathic scoliosis; BrAIST: bracing for adolescent idiopathic scoliosis trial; SRS: Scoliosis Research Society

## Competing interests

We declare that we have no competing interests'.

## Authors’ contributions

YK, MM and YN participated in the design of the study and helped to collect data.

TM conceived of the study, participated in its design, performed the statistical analysis and helped to draft the manuscript.

All authors read and approved the final manuscript.

## Declarations

Publication charges for this collection were funded by IRSSD 2014 Sapporo.

This article has been published as part of *Scoliosis* Volume 10 Supplement 2, 2015: Research into Spinal Deformities 9: Short Papers from the IRSSD 2014 Meeting. The full contents of the supplement are available online at http://www.scoliosisjournal.com/supplements/10/S2.
